# Negative Health Comparisons Decrease Affective and Cognitive Well-Being in Older Adults. Evidence from a Population-Based Longitudinal Study in Germany

**DOI:** 10.3389/fpsyg.2016.00999

**Published:** 2016-06-28

**Authors:** André Hajek, Hans-Helmut König

**Affiliations:** Department of Health Economics and Health Services Research, Hamburg Center for Health Economics, University Medical Center Hamburg-EppendorfHamburg, Germany

**Keywords:** health comparison, subjective well-being, positive affect, negative affect, life satisfaction, asymmetric effect, SWLS, PANAS

## Abstract

**Purpose:** To examine the effect of health comparisons on affective (AWB) and cognitive well-being (CWB) in older adults longitudinally.

**Methods:** Data were derived from the third and fourth wave of the German Ageing Survey (DEAS) which is a population-based prospective cohort study of community-dwelling subjects in Germany aged 40 and above (with 8,277 observations in fixed effects regressions). Health comparisons were assessed by the question “How would you rate your health compared with other people your age” (Much better; somewhat better; the same; somewhat worse, much worse). While AWB was quantified by using the Positive and Negative Affect Schedule (PANAS), CWB was assessed by using the Satisfaction with Life Scale (SWLS). Fixed effects regressions were used to analyze the effect of health comparisons on AWB and CWB.

**Results:** While positive health comparisons only slightly increased CWB (total sample), negative health comparisons markedly decreased CWB (total sample and women), and negative affects (women). Neither positive nor negative health comparisons affected positive affects.

**Conclusions:** Our findings stress the importance of negative health comparisons for CWB and negative affects in women. Comparison effects are asymmetric and in most cases upwards. Consequently, designing interventions to avoid upwards health comparisons might be a fruitful approach in order to maintain AWB and CWB.

## Introduction

In 1974, Richard Easterlin published a widely cited and famous study about the relation between subjective well-being and monetary growth. According to his famous Easterlin-Paradox richer individuals have a higher subjective well-being (SWB) than poorer individuals at a certain point in time (Easterlin, 1995). However, in industrialized countries increases in income over time do not increase SWB in the long run. This paradox is often explained by the assumption that SWB is affected by income in relative terms rather than in absolute terms. This means that the perception of individual income might be mainly affected by the income in comparison to other individuals (reference group, e.g., individuals of the same age cohort)—the “comparison income.”

Most of the previous studies in industrialized countries found that individuals have a markedly lower SWB when their income is lower than the income of a reference group. Contrarily, individuals with an income exceeding the income of a given reference group usually do not report a better SWB (Duesenberry, 1949; Holländer, 2001; Blanchflower and Oswald, 2004; Ferrer-i-Carbonell, 2005; Frijters et al., 2011; Maennig and Wilhelm, 2012). Therefore, income comparisons seems to be mainly upwards. Moreover, some of these studies found that the effect of upwards comparisons is more pronounced in men. This idea of asymmetric effects was introduced by Duesenberry (1949). This idea was also confirmed by Kahneman and Tversky as part as their prospect theory (Kahneman and Tversky, 1979). By using thought experiments, they found that you are angrier about losing $100 than you are satisfied about gaining $100. Baumeister et al. (2001) concludes that bad events are more powerful than good events.

While the existing studies focused on the impact of income comparisons on SWB, we hypothesize that the idea of comparisons affecting SWB is more general and therefore can be extended to the domain of health. Consequently and quite analogously, we hypothesize that the perception of self-rated health is mainly affected by the own health compared to the health of other individuals (for example, individuals in the same age cohort). We refer to it as “health comparisons.” According to the idea of asymmetric comparison effects, we hypothesize that health comparisons are mostly upwards. Based on the aforementioned studies that found gender differences in the effect of income comparisons on SWB, we hypothesize that gender differences in the effect of health comparisons on SWB exist. Consequently, it is assumed that health comparisons have a stronger impact on our outcome measures in men. Especially upwards comparisons might have a strong impact on SWB in men.

SWB can be defined as the individuals' current evaluation of their lives. It has two major components, affective well-being (AWB) and cognitive well-being (CWB). CWB refers to the cognitive evaluation of the whole life, whereas AWB refers to the experience of positive affects (e.g., joy, PA) and the absence of negative affects (e.g., anxiety, NA). AWB and CWB are different constructs, have different predictors (Kahneman and Deaton, 2010) and differ in their stability (Eid and Diener, 2004).

No study has examined the impact of health comparisons on CWB and AWB thus far. Consequently, our study provides first insights in the relationship between health comparisons and CWB as well as AWB using a representative sample of community-dwelling individuals aged 40 and above in Germany longitudinally. This knowledge is important to determine whether the idea of income comparisons can be extended to the domain of health. Furthermore, this knowledge is crucial to generate interventions (deliberate downwards comparisons; avoid upwards comparisons) which might be fruitful to maintain or increase AWB or CWB.

## Methods

### Sample

We used data from the third (year 2008) and fourth wave (year 2011) from the public release of the German Ageing Survey (DEAS). It is provided by the Research Data Centre of the German Centre of Gerontology (DZA). The German Ageing Survey is a representative survey of community-dwelling individuals aged 40 and above in Germany. Individuals were interviewed at home by trained staff. To this end, a standardized questionnaire was used. National probability sampling was done. 8,200 individuals participated in the third wave, whereas 4,855 individuals were interviewed in the fourth wave. Thereof, 2,864 individuals participated in the third and fourth waves and filled out the AWB and CWB items. The differences in the sample sizes between the third and fourth wave can mostly be explained by the introduction of new samples in the third wave. More insights with regard to the sampling frame and the composition of the sample were provided elsewhere (Engstler and Motel-Klingebiel, 2010). Informed consent was obtained from all individual participants included in the study.

### Affective and cognitive well-being

The Satisfaction with Life Scale (Pavot and Diener, 1993) with five items, each on a 5-point rating scale (index score from 1 to 5, high values indicate high *CWB*) was used to quantify life satisfaction (CWB). Furthermore, the Positive and Negative Affect Schedule (Watson et al., 1988) was used to assess *PA* and *NA*, each quantified with 10 items on 5-point rating scales, ranging from 1 (very slightly or not at all) to 5 (extremely). High values indicate high NA or PA. While Cronbach's Alpha was 0.87 for PA, it was 0.86 for NA. Moreover, Cronbach's Alpha was 0.89 for SWLS.

### Health comparisons

By using the question “How would you rate your health compared with other people your age” (Much better; somewhat better; the same; somewhat worse; much worse) *health comparisons* were assessed. The category “the same” was used as reference category.

### Control variables

We controlled for numerous time-dependent regressors which are assumed to be important for AWB and CWB. This is important to isolate the impact of health comparisons on AWB and CWB. For example, health comparisons might be related with other predictors such as self-assessed health or morbidity.

Consequently, we controlled for *family status* (Ref.: married, living together with spouse; married, living separated from spouse; divorced; widowed; never married), *age*, (log) *monthly household net income* in Euro, *employment status* (Ref.: working; retired; other: not employed), and *region*.

Furthermore, *morbidity* was taken into account (total number of physical diseases (0 if not present and 1 if present), actually ranging from 0 to 10 in the population studied. Physical diseases are as follows: high cholesterol; diabetes, high blood sugar levels; high blood pressure; heart attack, angina pectoris; cardiac insufficiency including coronary artery disease; stroke; circulatory disorders in the brain; circulatory disorders in the legs; joint degeneration (arthrosis) of the hips, knees, or spine; osteoporosis; inflammatory joint or spinal disease (arthritis or rheumatoid arthritis); chronic pulmonary disease (e.g., chronic bronchitis, pulmonary emphysema); cancer, malignant tumor (including leukemia); Stomach ulcer, intestinal ulcer; incontinence; Parkinson's disease; glaucoma or macular degeneration. These physical diseases were informed by the Charlson Comorbidity Index (Charlson et al., 1994). Additionally, *self-efficacy and optimism* (HOPE scale; Snyder et al., 1991), from 1 to 4 (high values indicate great self-efficacy) and the *number of important people in regular contact* (0–9) was taken into account. Cronbach's Alpha for the HOPE scale was 0.82. Moreover, the effect of *subjective health* (1 to 5, high values indicate bad subjective health) was taken into account to rule out that changes in health comparisons only reflect changes in subjective health.

For descriptive purposes, the time-constant sociodemographic variable *education* (level of education by ISCED-97 (International Standard Classification of Education; UNESCO, 2006) was used. It has three categories: low (0–2), medium (3–4), and high (5–6). As stated in the next chapter, time-constant variables cannot be included as independent variables in FE regressions.

### Statistical analysis

Longitudinal regression techniques offer the advantage of controlling for time-constant unobserved factors such as genetic disposition. This is crucial in SWB research since time-constant unobserved factors are often correlated with the predictors (Ferrer-i-Carbonell and Frijters, 2004). If this is the case, random effects (RE) regression techniques lead to biased (inconsistent) estimates (Cameron and Trivedi, 2005). Contrarily, FE regressions lead to consistent estimates in such a case (under the assumption of strict exogeneity). In contrast to RE regressions, which incorporate between- and within-variations, FE regressions solely exploit variations within individuals over time. Thus, the FE estimator is also called “Within estimator.” For this reason, solely time-dependent variables can be included in FE regression models. It is worth mentioning that cluster-robust standard errors were computed (Stock and Watson, 2008). Statistical analysis was conducted using Stata Release 14 (Stata Corp., College Station, Texas).

## Results

### Descriptive statistics

Descriptive statistics over time (wave 3 and wave 4) are depicted in Table 1. At wave 3, mean age was 62.3 years (±10.8 years), ranging from 40 to 93 years. Most of the individuals were male (50.4%), had medium education (50.2%), were married, living together with spouse (75.5%), and were retired (50.9%). The mean monthly household net income was €2,722.6 (±€2,929.9). While the mean self-efficacy (HOPE scale) was 3.1 (±0.4), the mean number of important people in regular contact was 4.8 (±2.8). Furthermore, mean self-assessed health was 2.4 (±0.8) and mean number of physical diseases was 2.3 (±1.8).

**Table 1:** **Descriptive statistics over time (Waves 3–4)**.

	**Wave 3 (*n* = 2,864)**	**Wave 4 (*n* = 2,864)**
Age: Mean (SD)	62.3 (10.8)	65.3 (10.8)
Female: N (%)	1,419 (49.6)	1,419 (49.6)
Marital Status: N (%)		
Married, living together with spouse	2,163 (75.5)	2,112 (73.8)
Married, living separated from spouse	32 (1.1)	35 (1.2)
Divorced	229 (8.0)	231 (8.1)
Widowed	288 (10.1)	337 (11.8)
Single	151 (5.3)	147 (5.1)
Level of Education (ISCED categories): N (%)		
Low (0-2)	192 (6.7)	192 (6.7)
Medium (3-4)	1,438 (50.2)	1,438 (50.2)
High (5-6)	1,233 (43.1)	1,233 (43.1)
Employment Status: N (%)		
Working	1,047 (36.6)	904 (31.6)
Retired	1,457 (50.9)	1,675 (58.6)
Other: not employed	359 (12.5)	281 (9.8)
Monthly household net income in Euro^a^: Mean (SD)	2,722.6 (2,929.9)	2,787.4 (2,154.9)
Number of important people in regular contact: Mean (SD)	4.8 (2.8)	5.0 (2.7)
Self-efficacy and optimism (HOPE Scale)^b^: Mean (SD)	3.1 (0.4)	3.0 (0.4)
Morbidity (total number of physical diseases)^c^: Mean (SD)	2.3 (1.8)	2.5 (1.8)
Self-assessed health^d^: Mean (SD)	2.4 (0.8)	2.5 (0.8)
Health Comparison: N (%)		
Much worse	65 (2.3)	74 (2.6)
Somewhat worse	268 (9.4)	251 (8.7)
The same	906 (31.6)	919 (32.1)
Somewhat better	1,177 (41.1)	1,177 (41.1)
Much better	448 (15.6)	443 (15.5)
PA (PANAS): Mean (SD)	3.6 (0.5)	3.5 (0.5)
NA (PANAS): Mean (SD)	2.0 (0.5)	2.0 (0.5)
CWB (SWLS): Mean (SD)	3.8 (0.7)	3.9 (0.7)

Missing values for metric variables (if occurred).

a 410 missing values in the third wave and 242 missing values in the fourth wave.

b 1 missing value in the third wave.

c 38 missing values in the third wave and 45 missing values in the fourth wave.

d 1 missing value in the third wave and 1 missing value in the fourth wave.

SD, Standard deviation.

As for our variable of main interest, health comparisons, most of the individuals rated their health as “the same” (31.6%) or “somewhat better” (41.1%) in comparison to other people their age. Mean PA was 3.6 (±0.5), mean NA was 2.0 (±0.5), and mean CWB was 3.8 (±0.7). Three years later (wave 4), the proportion of retired individuals rose to 58.6%. Besides, the other variables remained almost the same.

### Correlations

In Tables 2–5, pairwise correlation in levels are depicted for main variables (self-assessed health, self-efficacy, morbidity, health comparisons, AWB, and CWB), separately for women and men as well as wave 3 and wave 4. Moreover, pairwise correlation in differences (for women and men) are displayed in Tables 6, 7.

**Table 2:** **Pairwise correlations in levels (men; wave 3)**.

	**PA (PANAS)**	**NA (PANAS)**	**CWB (SWLS)**	**Self-assessed health**	**Morbidity (total number of physical diseases)**	**Self-efficacy and optimism (HOPE Scale)**	**Much better**	**Somewhat better**	**Somewhat worse**	**Much worse**
PA (PANAS)	1.00									
NA (PANAS)	−0.23^***^	1.00								
CWB (SWLS)	0.45^***^	−0.39^***^	1.00							
Self-assessed health	−0.37^***^	0.19^***^	−0.32^***^	1.00						
Morbidity (total number of physical diseases)	−0.23^***^	0.28^***^	−0.16^***^	0.42^***^	1.00					
Self-efficacy and optimism (HOPE Scale)	0.62^***^	−0.35^***^	0.62^***^	−0.31^***^	−0.19^***^	1.00				
Much better	0.19^***^	−0.10^***^	0.15^***^	−0.31^***^	−0.13^***^	0.17^***^	1.00			
Somewhat better	0.07^*^	−0.03	0.09^***^	−0.18^***^	−0.06^+^	0.07^*^	−0.33^***^	1.00		
Somewhat worse	−0.14^***^	0.10^***^	−0.16^***^	0.32^***^	0.17^***^	−0.13^***^	−0.14^***^	−0.28^***^	1.00	
Much worse	−0.17^***^	0.10^***^	−0.22^***^	0.37^***^	0.09^***^	−0.19^***^	−0.08^**^	−0.15^***^	−0.07^*^	1.00
Observations	2,572									

+ p < 0.10,

* p < 0.05,

** p < 0.01,

*** p < 0.001.

**Table 3:** **Pairwise correlations in levels (men; wave 4)**.

	**PA (PANAS)**	**NA (PANAS)**	**CWB (SWLS)**	**Self-assessed health**	**Morbidity (total number of physical diseases)**	**Self-efficacy and optimism (HOPE Scale)**	**Much better**	**Somewhat better**	**Somewhat worse**	**Much worse**
PA (PANAS)	1.00									
NA (PANAS)	−0.31^***^	1.00								
CWB (SWLS)	0.45^***^	−0.42^***^	1.00							
Self-assessed health	−0.30^***^	0.20^***^	−0.30^***^	1.00						
Morbidity (total number of physical diseases)	−0.24^***^	0.23^***^	−0.16^***^	0.45^***^	1.00					
Self-efficacy and optimism (HOPE Scale)	0.62^***^	−0.38^***^	0.58^***^	−0.26^***^	−0.16^***^	1.00				
Much better	0.12^***^	−0.08^*^	0.12^***^	−0.27^***^	−0.11^***^	0.13^***^	1.00			
Somewhat better	0.08^*^	−0.05	0.08^*^	−0.20^***^	−0.07^+^	0.05	−0.35^***^	1.00		
Somewhat worse	−0.09^**^	0.08^*^	−0.13^***^	0.32^***^	0.13^***^	−0.09^**^	−0.13^***^	−0.27^***^	1.00	
Much worse	−0.14^***^	0.11^***^	−0.18^***^	0.30^***^	0.10^***^	−0.14^***^	−0.06	−0.14^***^	−0.05	1.00
Observations	1,772									

+ p < 0.10,

* p < 0.05,

** p < 0.01,

*** p < 0.001.

**Table 4:** **Pairwise correlations in levels (women; wave 3)**.

	**PA (PANAS)**	**NA (PANAS)**	**CWB (SWLS)**	**Self-assessed health**	**Morbidity (total number of physical diseases)**	**Self-efficacy and optimism (HOPE Scale)**	**Much better**	**Somewhat better**	**Somewhat worse**	**Much worse**
PA (PANAS)	1.00									
NA (PANAS)	−0.22^***^	1.00								
CWB (SWLS)	0.49^***^	−0.38^***^	1.00							
Self−assessed health	−0.39^***^	0.16^***^	−0.35^***^	1.00						
Morbidity (total number of physical diseases)	−0.24^***^	0.23^***^	−0.19^***^	0.46^***^	1.00					
Self−efficacy and optimism (HOPE Scale)	0.64^***^	−0.36^***^	0.62^***^	−0.34^***^	−0.25^***^	1.00				
Much better	0.19^***^	−0.07^+^	0.13^***^	−0.31^***^	−0.11^***^	0.15^***^	1.00			
Somewhat better	0.08^**^	−0.02	0.12^***^	−0.19^***^	−0.08^*^	0.09^***^	−0.31^***^	1.00		
Somewhat worse	−0.11^***^	0.09^***^	−0.17^***^	0.33^***^	0.17^***^	−0.10^***^	−0.14^***^	−0.26^***^	1.00	
Much worse	−0.18^***^	0.10^***^	−0.21^***^	0.39^***^	0.15^***^	−0.18^***^	−0.08^**^	−0.15^***^	−0.07^+^	1.00
Observations	2,338									

+ p < 0.10,

* p < 0.05,

** p < 0.01,

*** p < 0.001.

**Table 5:** **Pairwise correlations in levels (women; wave 4)**.

	**PA (PANAS)**	**NA (PANAS)**	**CWB (SWLS)**	**Self-assessed health**	**Morbidity (total number of physical diseases)**	**Self-efficacy and optimism (HOPE Scale)**	**Much better**	**Somewhat better**	**Somewhat worse**	**Much worse**
PA (PANAS)	1.00									
NA (PANAS)	−0.31^***^	1.00								
CWB (SWLS)	0.44^***^	−0.46^***^	1.00							
Self−assessed health	−0.29^***^	0.17^***^	−0.28^***^	1.00						
Morbidity (total number of physical diseases)	−0.22^***^	0.21^***^	−0.21^***^	0.43^***^	1.00					
Self−efficacy and optimism (HOPE Scale)	0.62^***^	−0.36^***^	0.55^***^	−0.24^***^	−0.19^***^	1.00				
Much better	0.18^***^	−0.14^***^	0.14^***^	−0.32^***^	−0.13^***^	0.17^***^	1.00			
Somewhat better	0.09^**^	−0.06	0.10^**^	−0.16^***^	−0.09^**^	0.08^*^	−0.34^***^	1.00		
Somewhat worse	−0.10^**^	0.07	−0.13^***^	0.30^***^	0.17^***^	−0.10^**^	−0.13^***^	−0.25^***^	1.00	
Much worse	−0.12^***^	0.09^**^	−0.15^***^	0.31^***^	0.07	−0.09^**^	−0.07	−0.13^***^	−0.05	1.00
Observations	1,723									

^+^p < 0.10,

* p < 0.05,

** p < 0.01,

*** p < 0.001.

**Table 6:** **Pairwise correlations in differences (men)**.

	**Δ PA (PANAS)**	**Δ NA (PANAS)**	**Δ CWB (SWLS)**	**Δ Self-assessed health**	**Δ Morbidity (total number of physical diseases)**	**Δ Self-efficacy and optimism (HOPE Scale)**	**Δ Much better**	**Δ Somewhat better**	**Δ Somewhat worse**	**Δ Much worse**
Δ PA (PANAS)	1.00									
Δ NA (PANAS)	−0.16^***^	1.00								
Δ CWB (SWLS)	0.32^***^	−0.30^***^	1.00							
Δ Self−assessed health	−0.16^***^	0.08^*^	−0.11^***^	1.00						
Δ Morbidity (total number of physical diseases)	−0.10^***^	0.25^***^	−0.15^***^	0.13^***^	1.00					
Δ Self−efficacy and optimism (HOPE Scale)	0.38^***^	−0.25^***^	0.47^***^	−0.11^***^	−0.13^***^	1.00				
Δ Much better	0.07	−0.01	0.07	−0.13^***^	−0.03	0.08	1.00			
Δ Somewhat better	−0.03	0.03	−0.03	−0.08^+^	−0.07	−0.06	−0.52^***^	1.00		
Δ Somewhat worse	−0.08	0.05	−0.03	0.20^***^	0.09^*^	−0.07	−0.04	−0.19^***^	1.00	
Δ Much worse	−0.02	−0.03	−0.04	0.08	0.06	0.01	−0.03	−0.05	−0.22^***^	1.00
Observations	2,146									

+ p < 0.10,

* p < 0.05,

^**^p < 0.01,

*** p < 0.001.

**Table 7:** **Pairwise correlations in differences (women)**.

	**Δ PA (PANAS)**	**Δ NA (PANAS)**	**Δ CWB (SWLS)**	**Δ Self-assessed health**	**Δ Morbidity (total number of physical diseases)**	**Δ Self-efficacy and optimism (HOPE Scale)**	**Δ Much better**	**Δ Somewhat better**	**Δ Somewhat worse**	**Δ Much worse**
Δ PA (PANAS)	1.00									
Δ NA (PANAS)	−0.17^***^	1.00								
Δ CWB (SWLS)	0.33^***^	−0.28^***^	1.00							
Δ Self−assessed health	−0.11^***^	0.08^*^	−0.11^***^	1.00						
Δ Morbidity (total number of physical diseases)	−0.08^*^	0.23^***^	−0.09^*^	0.13^***^	1.00					
Δ Self−efficacy and optimism (HOPE Scale)	0.39^***^	−0.22^***^	0.41^***^	−0.10^***^	−0.06	1.00				
Δ Much better	0.06	−0.00	0.05	−0.16^***^	−0.02	0.01	1.00			
Δ Somewhat better	0.02	−0.04	0.03	−0.06	−0.00	0.05	−0.50^***^	1.00		
Δ Somewhat worse	0.00	0.08	−0.03	0.14^***^	0.00	−0.05	−0.03	−0.14^***^	1.00	
Δ Much worse	−0.07	0.08	−0.13^***^	0.14^***^	−0.03	−0.07	−0.03	−0.03	−0.24^***^	1.00
Observations	2,068									

^+^p < 0.10,

* p < 0.05,

^**^p < 0.01,

*** p < 0.001.

### Regression analysis

Findings of the FE are depicted in Table 8. For the sake of space, region, marital status and employment status were not shown in this table (but these results are available upon request).

**Table 8:** **Longitudinal predictors of negative affect (NA), positive affect (PA), and life satisfaction (CWB): Results of fixed effects regressions (Waves 3-4) in the total sample, men, and women**.

**Independent variables**	**(1) NA—Total Sample**	**(2) NA—Men**	**(3) NA—Women**	**(4) PA—Total Sample**	**(5) PA—Men**	**(6) PA—Women**	**(7) CWB—Total Sample**	**(8) CWB—Men**	**(9)CWB—Women**
Age	−0.0101^**^	−0.00603	−0.0122^*^	−0.00319	−0.00348	−0.00397	0.0204^***^	0.0255^***^	0.0133^*^
	(0.00340)	(0.00440)	(0.00518)	(0.00308)	(0.00417)	(0.00462)	(0.00405)	(0.00532)	(0.00626)
(Log) Monthly household net income in Euro	0.0425	0.0306	0.0325	0.113^**^	0.154^*^	0.0758+	0.0765+	0.0921	0.0486
	(0.0377)	(0.0520)	(0.0541)	(0.0370)	(0.0602)	(0.0453)	(0.0428)	(0.0633)	(0.0576)
Number of important people in regular contact	0.00141	−0.00181	0.00550	0.00638^*^	0.00329	0.0101^*^	0.000143	−5.67e−05	−6.26e−05
	(0.00307)	(0.00412)	(0.00455)	(0.00271)	(0.00345)	(0.00430)	(0.00364)	(0.00472)	(0.00561)
Self−efficacy and optimism (HOPE Scale)	−0.295^***^	−0.280^***^	−0.293^***^	0.468^***^	0.438^***^	0.501^***^	0.646^***^	0.678^***^	0.603^***^
	(0.0360)	(0.0515)	(0.0485)	(0.0314)	(0.0421)	(0.0473)	(0.0408)	(0.0509)	(0.0627)
Self−assessed health	0.0280^*^	0.0411^*^	0.0111	−0.0403^**^	−0.0365^*^	−0.0437^*^	−0.0145	−0.0130	−0.0136
	(0.0139)	(0.0181)	(0.0207)	(0.0128)	(0.0165)	(0.0199)	(0.0171)	(0.0211)	(0.0273)
Health comparison: Much better (Ref.: The same)	0.0187	0.0572	−0.0304	0.0208	−0.00338	0.0460	0.0669^*^	0.0749	0.0671
	(0.0280)	(0.0358)	(0.0433)	(0.0273)	(0.0356)	(0.0413)	(0.0339)	(0.0461)	(0.0498)
Health comparison: Somewhat better	0.0186	0.0525+	−0.0234	−0.00148	−0.0169	0.0139	0.00521	0.00284	0.0214
	(0.0198)	(0.0274)	(0.0288)	(0.0177)	(0.0241)	(0.0266)	(0.0222)	(0.0298)	(0.0333)
Health comparison: Somewhat worse	0.0625+	0.0331	0.102^*^	−0.0183	−0.0689+	0.0273	−0.0674+	−0.0498	−0.0920+
	(0.0325)	(0.0422)	(0.0478)	(0.0303)	(0.0411)	(0.0436)	(0.0375)	(0.0540)	(0.0525)
Health comparison: Much worse	0.0386	−0.230	0.292^**^	−0.0712	−0.0330	−0.0933	−0.341^***^	−0.155	−0.534^***^
	(0.0979)	(0.153)	(0.104)	(0.0751)	(0.107)	(0.104)	(0.0967)	(0.133)	(0.129)
Morbidity (total number of physical diseases)	0.0509^***^	0.0517^***^	0.0560^***^	−0.00917	−0.00731	−0.0127	−0.0196^*^	−0.0197+	−0.0220+
	(0.00738)	(0.00946)	(0.0112)	(0.00701)	(0.00871)	(0.0111)	(0.00870)	(0.0115)	(0.0130)
Constant	2.754^***^	2.827^***^	3.394^***^	1.046^**^	0.687	1.643^***^	−0.0165	−0.284	0.797
	(0.383)	(0.569)	(0.496)	(0.361)	(0.547)	(0.465)	(0.450)	(0.669)	(0.569)
Observations	8,277	4,277	3,996	8,276	4,276	3,996	8,277	4,276	3,997
Number of Individuals	6,052	3,112	2,937	6,051	3,111	2,937	6,052	3,110	2,939
*R*^2^	0.094	0.103	0.117	0.165	0.164	0.177	0.189	0.215	0.174

Beta-Coefficients were reported; Cluster-robust standard errors in parentheses; Regressions are also controlled for region, marital status, and employment status;

*** p < 0.001,

** p < 0.01,

* p < 0.05,

+ p < 0.10;

Observations with missing values were dropped (listwise deletion). While CWB was measured by using the Satisfaction with Life Scale (SWLS), AWB (PA; NA) was quantified by using the Positive and Negative Affect Schedule (PANAS).

When the own health compared with other people in the same age was initially rated as “the same” and subsequently rated as “somewhat worse” or “much worse” (or vice versa), we refer to it as “negative health comparisons.” “Positive health comparisons” were defined analogously. Consequently, “the same” was used as reference category.

In the total sample, negative or positive health comparisons did not affect NA. Gender specific regression analysis showed that negative health comparisons significantly and markedly increased NA in women (“somewhat worse”: β = 0.10; “much worse”: β = 0.29). As for control variables, NA significantly increased with less self-efficacy (HOPE scale) and higher comorbidity in the total sample and in both sexes. Furthermore, NA increased with decreasing age in the total sample and in women as well as with worse subjective health in the total sample and in men.

The occurrence of negative or positive health comparisons did not affect PA in the total sample and in both sexes. PA increased with more self-efficacy and better subjective health in the total sample and in both sexes. Moreover, PA increased with higher income in the total sample and in men as well as with the number of important people in regular contact in the total sample and in women.

While positive health comparisons (“much better”: β = 0.07) significantly but slightly increased CWB in the total sample, negative health comparisons markedly decreased CWB in the total sample (“much worse”: β = −0.34) and in women (“much worse”: β = −0.53). Furthermore, increasing age and more self-efficacy increased CWB in the total sample and in both sexes. Moreover, increasing comorbidity decreased CWB in the total sample. It is worth noting that subjective health was not significantly related with CWB.

It is also worth mentioning that there was a significant interaction between gender and the health comparison status “much worse” for NA (*p* < 0.01) and CWB (*p* < 0.05).

## Discussion

### Main findings

While positive health comparisons only slightly increased CWB (total sample), negative health comparisons markedly decreased CWB (total sample and women) and NA (women). Neither positive nor negative health comparisons affected PA.

### Previous research

Since this is the first study investigating the impact of health comparisons on AWB and CWB, our findings are difficult to compare with recent studies using income comparisons as independent variable. Some of these previous studies found that individuals weight upward *income* comparisons more heavily than downward comparisons using micro datasets (Ferrer-i-Carbonell, 2005; Boyce et al., 2010). These studies used measures of happiness as outcome variables. However, even if it is assumed that the concept of comparisons is a broad one and is therefore not limited to income comparisons, it remains an open question to what degree income and health comparisons are comparable.

Interestingly, in our study solely *women* were affected by *negative* health comparisons (NA and CWB), conforming the idea of asymmetric effects which was introduced by Duesenberry (1949) and corroborated by Kahneman and Tversky (1979) as well as Baumeister et al. (2001). Regarding income comparisons, recent studies found *upward* income comparison effects mostly in *men*. Differences between these results and our findings might be explained by aforementioned differences between income and health comparisons. More specifically, contrarily to income, health is a non-monetary parameter. Some studies have found that income has a high relevance for men, whereas women weight some non-monetary variables such as family-related factors more heavily (Clark et al., 2008; Clark and Georgellis, 2013). However, thus far, it remains an open question whether gender differences exist in the long-term effect of health-related predictors on SWB. In sum, we assume that (negative) health comparisons are important for the SWB of women, while men—as already found by recent studies—seem to be more heavily affected by (negative) income comparisons.

In our view, health comparisons in women can be seen as hygiene factor (Herzberg, 1966)—a famous term in job satisfaction research. Hygiene factors are factors that do not lead to higher satisfaction if they are present, although dissatisfaction results from their absence such as job security, work conditions or vacations in job satisfaction research–or health comparisons in AWB and CWB research.

Extending previous research, we also examined AWB (NA and PA). For example, Luhmann et al. (2012) found in a meta-analysis that life events (such as unemployment, migration, marriage, child birth or divorce) have very different effects on AWB and CWB. Furthermore, Tay and Diener (2011) found that positive feelings (“smile/laugh” and “enjoyment”) were most related with social and respect needs. Furthermore, negative feelings (“worry,” “sadness,” “depression,” and “anger”) were most associated with autonomy, respect and basic needs (such as basic needs for food and shelter), whereas life evaluation was most associated with basic needs. It is worth noting that their outcome measures are strongly related to our outcome variables. Moreover, one could argue that negative health comparisons are related to the concept of basic needs. Thus, the strong impact of negative health comparisons on CWB are in line with their findings. For more information on needs, please see Deci and Ryan (2000) as well as Ryff and Keyes (1995).

In contrast to NA, it is quite interesting that health comparisons did not affect PA in our study. We assume that negative health comparisons might amplify negative emotions, whereas these negative comparisons did not affect positive feelings which should be further investigated in future studies.

### Strengths and limitations

This is the first study investigating the effect of health comparisons on *affective* and *cognitive well-being* in Germany using a longitudinal approach. Additionally, by using FE regressions (1) insights into the mechanisms can be deduced and (2) time-constant unobserved heterogeneity was taken into account. Consequently, the estimates are consistent (under the assumption of exogeneity). Another major strength is that data were derived from a population-based study of community-dwelling individuals aged 40 and above in Germany. Furthermore, it is worth highlighting that we used validated measures to assess PA as well as NA (PANAS) and CWB (SWLS).

However, it should be taken into account that simultaneity bias (from SWB to health comparisons) cannot be ruled out. Furthermore, the association between SWB and health comparisons might be explained by time-dependent third variables. Furthermore, it should be taken into consideration that the reference group for health comparisons was stated explicitly (age bracket). However, other reference groups such as colleagues or good friends might also affect the health comparison process (Roberts, 1999). For example, individuals might compare their own health with individuals in the same age bracket, close friends and colleagues. Nevertheless, it is assumed that the age-bracket is the key and most salient dimension for health comparisons. Moreover, it should be taken into account that our estimates might be biased downwards for reasons of panel attrition in the German Ageing Survey (Schiel et al., 2011). Therefore, it was tested whether differences in health comparisons exist between individuals who participated in (i) the third and fourth wave and individuals who participated in (ii) the third wave. Actually, individuals with complete data had more positive health comparisons than the other group. Consequently, it is assumed that our estimates are somewhat biased downwards.

Furthermore, for reasons of data availability, personality measures were not controlled. Thus, we cannot rule out that these factors (such as neuroticism or extraversion) affect the relationship between health comparisons and our outcome measures.

Besides, it is worth emphasizing, that negative health comparisons are associated with lower SWB at zero-order correlation in men (Tables 2, 3). After adjusting for the potential confounders in regression analysis, negative health comparisons become insignificant in men. Thus, the non-significant impact of health comparisons on the SWB in men might be explained by including control variables important for men, whereas the impact of health comparisons on the SWB in women might be explained by the fact that control variables important for women were not entered in the model.

## Conclusion and future research

Our findings stress the importance of negative health comparisons for CWB and NA in older women. Data suggest that health comparison effects are asymmetric and in most cases upwards. Moreover, it seems that the concept of (asymmetric) comparisons can be extended to the domain of health.

Designing interventions to avoid upwards health comparisons might be a fruitful approach in order to maintain AWB and CWB (Buunk et al., 1990; Micari and Pazos, 2014). As far as data are available, future studies should incorporate personality which may moderate the relationship (Boyce and Wood, 2011; Proto and Rustichini, 2015). Moreover, factors such as self-esteem should be taken into consideration (Crocker et al., 1987). Furthermore, another assessment of CWB was recently suggested (life evaluation, Tay and Diener, 2011). This should be included in future studies (e.g., by using the Cantril Self-Anchoring Striving Scale (Cantril, 1965).

## Author contributions

Substantial contributions to the conception or design of the work; or the acquisition, analysis, or interpretation of data for the work: AH, HHK. Drafting the work or revising it critically for important intellectual content: AH, HHK. Final approval of the version to be published: AH, HHK. Agreement to be accountable for all aspects of the work in ensuring that questions related to the accuracy or integrity of any part of the work are appropriately investigated and resolved: AH, HHK.

### Conflict of interest statement

The authors declare that the research was conducted in the absence of any commercial or financial relationships that could be construed as a potential conflict of interest.
